# Assessing the Impact of an Advanced Clinical Decision Support System on Medication Safety and Hospital Readmissions in an Innovative Transitional Care Model: A Pilot Study

**DOI:** 10.3390/jcm11082070

**Published:** 2022-04-07

**Authors:** Jennifer M. Bingham, Lindsey Baugham, Andriana Hilaneh, Karley Tranchina, Daniel Arku, Becka Eckert, Nicole Scovis, Jacques Turgeon

**Affiliations:** 1 Tabula Rasa HealthCare, Office of Translational Research & Residency Programs, 228 Strawbridge Dr, Moorestown, NJ 08057, USA; jbingham@trhc.com (J.M.B.); nscovis@trhc.com (N.S.); 2 College of Pharmacy, University of Arizona, Tucson, AZ 85721, USA; lbaugham@pharmacy.arizona.edu (L.B.); ahilaneh@pharmacy.arizona.edu (A.H.); ktranchina@pharmacy.arizona.edu (K.T.); arku@pharmacy.arizona.edu (D.A.); beckert@pharmacy.arizona.edu (B.E.); 3 Tabula Rasa HealthCare, Precision Pharmacotherapy Research & Development Institute, 13485 Veterans Way, Orlando, FL 32827, USA; 4 Faculty of Pharmacy, Universite de Montreal, Pavillon Jean-Coutu, 2940, Chemin de la Polytechnique, Montreal, QC H3T IJ4, Canada

**Keywords:** transition of care, clinical decision support system, pharmacist

## Abstract

(1) Background: Adverse drug events and inappropriate use of medications lead to hospitalizations, medication-related morbidity, and mortality. This study examined whether a novel medication risk prediction tool, the MedWise Risk Score™, was associated with medication safety-related problem (MRP) identification and whether integration into an existing innovative transitions of care (TOC) service could decrease readmissions. (2) Methods: This retrospective comparator group study assessed patients discharged from a hospital in southern Arizona between January and December 2020. Participants were included in the study if they were 18 years of age or older, referred to the pharmacist for TOC services, and received a pharmacist consultation within one-week post discharge. Patients were categorized into two groups: (1) medication safety review (MSR)-TOC service (intervention) or (2) existing innovative TOC service (control). (3) Results: Of 164 participants, most were male (57%) and were between 70–79 years of age. Overall, there were significantly more drug-drug interactions (DDI) MRPs identified per patient in the intervention vs. control group for those who were readmitted (3.7 ± 1.5 vs. 0.9 ± 0.6, *p* < 0.001) and those who were not readmitted (2 ± 1.3 vs. 1.3 ± 1.2, *p* = 0.0120). Furthermore, of those who were readmitted, the average number of identified MRPs per patient was greater in the intervention group compared to the control (6.3 vs. 2.5, respectively, *p* > 0.05). Relative to the control, the readmission frequency was 30% lower in the treatment group; however, there was insufficient power to detect significant differences between groups. (4) Conclusions: The integration of a medication risk prediction tool into this existing TOC service identified more DDI MRPs compared to the previous innovative TOC service, which lends evidence that supports its ability to prevent readmissions. Future work is warranted to demonstrate the longitudinal impact of this intervention in a larger sample size.

## 1. Introduction

The inappropriate use of medication increases the risk of adverse drug events (ADEs) [1]. While ADEs are associated with an increased risk of hospital readmission [2,3], studies also suggest that most events are predictable and preventable [4,5,6], particularly through the integration of transitional care services [7,8] and the use of medication risk prediction tools [6].

The MedWise™ Risk Score (MRS) is a novel medication risk prediction tool that could help mitigate the risk of readmission due to ADEs. The MRS identifies patients at risk of an ADE [6] and works in tandem with an advanced clinical decision support software (CDSS)—MedWise—that helps pharmacists to identify clinically relevant medication-related problems (MRPs) [6,9]. An aggregate score is computed strictly from a medication regimen’s pharmacokinetic and pharmacodynamic characteristics. Specifically, the MRS quantifies, and aggregates risks associated with competitive inhibition, sedative burden, anticholinergic burden, and drug-induced long QT syndrome [9,10,11]. To date, others have validated that an elevated MRS is associated with several negative outcomes, which include ADEs, emergency department visits, hospitalizations, medical expenditures, and mortality [12,13]. Regarding readmission, San Filippo et al. found that the MRS can preemptively identify patients at the highest risk of hospital readmission within 30 days post discharge [14]. Nevertheless, it is unknown whether the MRS can help pharmacists identify relevant MRPs during the transition from hospital to home, which could help reduce readmissions.

To address this gap in the literature, a retrospective study was developed to evaluate the use of the MRS™ in identifying medication safety-related problems and reduce hospital readmissions on top of an existing transition of care (TOC) service at a local hospital in Southern Arizona.

## 2. Materials and Methods

### 2.1. Study Design

This retrospective review included data collected between January 2020 and December 2020. Participants were adult patients discharged from a local Southern Arizona hospital. This retrospective review was approved by the Institutional Review Board (approved 23 April 2020; protocol No. 2004557218).

### 2.2. Context and Setting

In 2015, a local hospital in Southern Arizona created an innovative, TOC program designed to address ADEs and mitigate 30-day all-cause readmissions [15]. It is unique from other TOC models given its sustainable model of interprofessional care, including transitional care coordinators, pharmacists, and nurses who use standardized clinical materials to provide a replicable service, regardless of which institution or clinical team is executing the program [8,15,16]. The care team works together to provide a personalized medication therapy management (MTM) TOC service aimed at reducing readmissions, improve patient health outcomes, and decrease costs for both the patient and hospital [15]. Since inception, it has proven to be an effective TOC service, as shown by steadily reduced readmission rates for patients at 30-, 60-, and 90-days post hospital discharge [15,17]; an average annual reduction of $3000 in Medicare beneficiary expenditures; and a steadily increased return-on-investment at 30- (32.94:1), 60- (68.20:1), and 90-days (77:59:1) post discharge [17].

### 2.3. Study Participants

Participants from January 2020 to December 2020 were included in the pilot study if they were 18 years of age or older, referred to the pharmacist for TOC services, and received a pharmacist consultation within one week post discharge. Patients were categorized into two groups based on: (1) participation in the MSR-TOC service (intervention) or (2) participation in the existing innovative TOC service (control).

### 2.4. Control

Telephonic consultations were conducted within one week post discharge, and a follow-up call was completed two weeks later. As previously described by Bingham et al., the normative TOC consultation involved [8]:

1. ***Medication reconciliation and chart review:*** The pharmacist performed a chart review prior to contacting the patient within one week of hospital discharge. They were expected to review laboratory values, provider notes, prior-to-admission medication lists, discharge medication orders, and the discharge summary. In addition, the pharmacist assessed for discrepancies and medications that were unintentionally not continued upon discharge.
2. ***Identification of MRPs:*** The pharmacist assessed the discharge medication list for appropriateness related to medication safety and access using a drug interaction screening software system (DISS) and clinical judgement. MRPs included drug-disease interactions, drug-drug interactions (DDIs), inappropriately dosed medications, patient-reported ADEs, and high-risk medications (HRM). Medication access concerns included barriers to medication adherence and financial or cost-related concerns.
3. ***Medication counseling:*** The pharmacist provided teach-back education on condition specific counseling to the patient. They also provided medication counseling on related discharge condition specific medications. Lastly, the pharmacist recorded their progress note in the hospital electronic health record with clinically relevant recommendations for outpatient providers, specialists, and/or dispensing pharmacies. A second follow-up consultation was performed two weeks later by the pharmacist.

### 2.5. Intervention

The intervention group received the same innovative TOC services as the control group. However, instead of investigating potential MRPs using a usual DISS, the specially trained pharmacist used the advanced CDSS (MedWise™) to calculate an MRS during the review. Table 1 provides an overview of the differences in DDI assessments between the control and intervention group.

Prior to providing direct patient care services, the pharmacist was required to successfully complete a certification program specific to the advanced CDSS. Next, the pharmacist assessed for MRPs using data displayed in the CDSS, which included risk predictions for pharmacokinetic drug interactions, sedative burden, anticholinergic burden, and drug-induced long QT syndrome. Pharmacists used these findings to craft interventions that would reduce the MRS and resolve the identified MRP(s).

### 2.6. Data Collection

Control and intervention data were collected for age, sex, readmission status 30 days post discharge, and readmission diagnosis. MRPs identified by the pharmacist during the initial consultation included the number of: (1) DDIs; (2) drug-disease interactions; (3) inappropriately dosed medications; (4) reported ADEs; and (5) high-risk medications (e.g., anticoagulants, insulin, digoxin).

Intervention data were collected for MRS (pre-pharmacist consultation, post-pharmacist consultation, post-provider review of pharmacist recommendations). Systematized Nomenclature of Medicine-Clinical Terms (SNOMED-CT codes) [18] were used to code recommendations made by the pharmacist during the consultation and post-provider review of pharmacist recommendations. SNOMED-CT options included recommendation to (1) change medication (428711000124105); (2) decrease medication dose (428791000124100); (3) increase medication dose (428811000124101); (4) change timing of medication administration (459221000124100); (5) start medication therapy (428861000124103); (6) discontinue medication (4701000124104); or (7) start medication monitoring (428871000124105) [18]. All patient data were deidentified, and access was limited to study investigators.

### 2.7. Study Outcomes and Analysis

The primary objective was the association of the novel MSR-TOC service on identifiable MRPs compared to those who received the existing TOC service using a two-sample t-test. Secondary outcomes included an exploratory investigation on the association between readmission rates and the type of intervention provided to the patient. Statistical analysis was performed to assess the association between MRPs and pharmacist interventions using an independent sample t-test. A Wilcoxon-rank sum test was used to compare the difference in MRS pre- and post-pharmacist intervention, as well as the difference in MRS post-pharmacist intervention and post-provider review of pharmacist interventions. A Fisher’s exact test was used to compare the difference in readmissions. All tests used an a priori alpha level of 0.05. Data analysis was conducted using Stata 16.

## 3. Results

The study sample consisted of 164 participants who received pharmacist services. The majority were 70–79 years of age (48%) and male (57%). Overall, 20% (*n* = 32) were randomly stratified into the intervention group and 80% (*n* = 132) into the control group (comparison). See Table 2 for details.

### 3.1. Medication Safety-Related Problems

Table 3 reports the number of MRPs identified per patient. There were three significant findings. First, pharmacists identified an average of 2.8 more DDIs per patient in the intervention group compared to the control for those who were readmitted (3.7 ± 1.5 vs. 0.9 ± 0.6, *p* < 0.001). Second, pharmacists identified more DDIs per patient among those who were not readmitted (2 ± 1.3 vs. 1.3 ± 1.2, *p* = 0.0120). Finally, the average number of identified MRPs per patient was greater in the intervention group compared to the control (4.0 ± 2.3 vs. 3.1 ± 2.3, *p* = 0.0430) for those who were not readmitted.

### 3.2. Hospital Readmissions

Compared to the control, a smaller proportion of intervention patients were readmitted within 30 days post discharge, which translated to a 30% relative decrease in the readmission frequency (9% vs. 13%). While we did not have enough statistical power to detect significant differences (*p* > 0.05) in all-cause readmissions, we did find that there were more readmissions caused by ADEs in the usual care group compared to the intervention group. See Table 4 for details.

### 3.3. Medication Risk Score and Clinical Interventions

In the intervention group, there was no difference in MRS between those who were readmitted versus those who were not for the pre-pharmacist intervention groups, post-pharmacist intervention groups, or post-provider review of recommendations. Of those who were readmitted, there was a slight increase in the median MRS post-provider review compared to post-pharmacist intervention, yet this increase was not significant. See Table 5 for details. A complete summary of pharmacists’ recommendations can be found in the appendix.

## 4. Discussion

In our small pilot study of 164 patients, we found that pharmacists who used an advanced CDSS—MedWise™—and ADE risk stratification tool—the MRS—were able to identify more total MRPs per patient compared to the existing TOC service. The increase in MRPs was largely due to DDIs. Specifically, pharmacists identified nearly three more DDIs per readmitted patient and nearly one more DDI per non-readmitted patient compared to the control. To resolve MRPs, pharmacists intervened to reduce the intervention group’s overall MRS, which reflected an attenuated ADE risk. Despite a similar number of all-cause readmissions between groups, no readmission was caused by probable ADEs in the intervention group, whereas 17% of the control’s readmissions were likely medication-induced. Collectively, our findings suggest that these advanced technologies can add value even when integrated into an already established, successful TOC model.

Our findings were in line with other literature that describes the impact these technologies have on pharmacist-driven interventions. First, higher risk scores increase the pharmacist’s capability of identifying more total MRPs (MRPs = 0.5 + 0.07 × MRS + 0.006 × MRS^2^) [6]. Additionally, DDIs have been found to be one of the most common types of MRPs identified by clinical pharmacists using the CDSS in medically complex older outpatients (37% of all MRPs) and in Medicare Part D beneficiaries (27% of all MRPs [6,19]. Thus, it is logical that we found a higher total number of MRPs—especially DDIs—in our intervention group. Specific to the TOC setting, a case study illustrated how these technologies helped a pharmacist identify and resolve several MRPs and DDIs as a way to reduce ADE-related readmission risks [20].

The design of the novel CDSS can explain the higher prevalence of MRPs and DDIs. First, the primary clinical visualization in the CDSS is a matrix-based assessment of each drug’s metabolic pathway (e.g., cytochrome P450 [CYP]). This permits pharmacists to assess pharmacokinetic drug interactions simultaneously, without redundant alerts. This is especially useful when the clinician is faced with complex polypharmacy, which traditionally makes DDI identification and interpretation more challenging [21]. Moreover, the CDSS accounts for other exhaustive pharmacokinetic and pharmacodynamic information related to each active ingredient, aiding in the identification of other MRPs related to sedative burden, anticholinergic burden, and drug-induced long QT syndrome. In addition, the CDSS can ingest patient-specific factors, such as age, sex, allergies, International Statistical Classification of Diseases (ICD)-10 code(s), and pertinent laboratory results. Given this comprehensive design, it likely afforded pharmacists in this study more opportunities to identify condition-specific contraindications and warnings related to DDIs compared to traditional DISS. This is important because others have demonstrated that about 20% of all ADE-related admissions are due to DDIs [22].

Regarding readmissions, our study was also consistent with other literature. First, several publications have demonstrated a 30% decrease in readmission after pharmacist-led TOC interventions, which was similar to what we observed [8,23]. Unfortunately, we were underpowered to detect this outcome. To reach statistical significance, our study should have needed 323 subjects. They are also consistent with another study that demonstrated an association between reduced MRS values and hospitalizations [14].

### Strengths and Limitations

The major strength of this study was that it was the first to evaluate the use of this advanced and novel CDSS in a TOC setting. As mentioned above, other studies have been predominately focused on pharmacist-led interventions for community-dwelling older adults.

There were some limitations as well. First, our small sample size minimized our power to detect readmissions. Second, patients were only followed for two weeks after the initial consultation, preventing the ability to draw long-term conclusions. Third, the investigators did not have ADE-specific readmission data. In addition, the study did not randomize patients. Rather, ADE-specific readmissions needed to be inferred based on admitting diagnoses that are commonly caused by medications. Finally, this study was only conducted at one hospital, thus limiting its generalizability to other settings and populations.

## 5. Conclusions

The integration of an advanced clinical decision support system and a medication risk predictive score into an existing interdisciplinary transitions of care service identified more MRPs—especially DDIs—and tended to reduce ADE-related readmissions compared to the existing innovative TOC service. Further studies should be performed with a larger number of subjects and in a prospective manner with a randomization design to confirm these observations.

## Figures and Tables

**Table 1:** Description of MRP assessments in intervention and control groups.

Control Group	Intervention Group
Pharmacist enters medication details into a drug interaction screening software system (DISS). The severity of the DDI interaction is calculated upon pharmacist entry of the following: ∘ Medication specific: ∘ name ∘ Patient specific: ∘ allergies Major and/or severe DDI are validated by the pharmacist using a secondary DISS.	Pharmacist enters medication details into MedWise™. The MRS is calculated in real time for every patient upon pharmacist entry of the following: ∘ Medication specific: ∘ name ∘ strength ∘ dose ∘ route of administration ∘ time of administration ∘ frequency ∘ Patient specific: ∘ age ∘ sex ∘ allergies ∘ International Statistical Classification of Diseases (ICD)-10 code(s) ∘ laboratory results ∘ QT interval results The MRS is displayed prominently at the top of the patient profile. Pharmacist reviews the drug interaction matrix for drug regimen alteration and recommendations, then makes an informed decision to reduce the MRS.

**Table 2:** Patient Characteristics.

Variable	Total *N* = 164	Intervention *N* *=* 32	Control *N* = 132	*p*-Value
Sex, *N* (%)				
Male	93 (57)	20 (63)	73 (55)	0.461
Female	71 (43)	12 (38)	59 (45)	0.461
Age, years (μ ± SD)	76 ± 7.2	77 ± 7.8	75 ± 7.0	0.277
50–59	55 ± 3.6	0	55 ± 3.6	-
60–69	67 ± 2.4	66 ± 2.9	67 ± 2.3	0.836
70–79	75 ± 2.8	75 ± 2.6	75 ± 2.8	0.971
80–89	84 ± 2.7	84 ± 2.8	84 ± 2.7	0.501

Note: Statistical analyses were performed using a *t*-test. An a priori alpha level of 0.05 was used.

**Table 3:** Medication safety-related problems identified by the pharmacist per patient.

Variable	Total *N* = 164 Mean ± SD	Intervention *N* = 32 Mean ± SD	Control *N* = 132 Mean ± SD	*p*-Value
**Readmitted (*N* = 20)**				
Drug-Disease Interactions	0.4 ± 0.6	1 ± 1	0.2 ± 0.4	0.3153
Drug-Drug Interactions	1.3 ± 1.3	3.7 ± 1.5	0.9 ± 0.6	<0.001
Inappropriately Dosed Medications	0.2 ± 0.4	0	0.2 ± 0.4	0.0826
Adverse Drug Reactions	0.3 ± 0.5	0.3 ± 0.6	0.3 ± 0.5	0.9197
High-risk Medications	1 ± 0.9	1.3 ± 0.6	0.9 ± 1	0.3282
Total MRPs Per Patient	3.1 ± 2.1	6.3 ± 2.3	2.5 ± 1.5	0.0913
**Not Readmitted (*N* = 144)**				
Drug-Disease Interactions	0.5 ± 0.6	0.5 ± 0.7	0.4 ± 0.6	0.5570
Drug-Drug Interactions	1.4 ± 1.3	2 ± 1.3	1.3 ± 1.2	0.0120
Inappropriately Dosed Medications	0.4 ± 0.6	0.2 ± 0.6	0.4 ± 0.7	0.2304
Adverse Drug Reactions	0.2 ± 0.5	0.4 ± 0.7	0.2 ± 0.4	0.0807
High-risk Medications	0.8 ± 0.8	0.9 ± 0.7	0.8 ± 0.9	0.6420
Total MRPs Per Patient	3.3 ± 2.3	4.0 ± 2.3	3.1 ± 2.3	0.0430

Note: Statistical analyses were performed to assess the association between MRPs and pharmacist interventions using an independent sample *t*-test. An a priori alpha level of 0.05 was used.

**Table 4:** Characteristics and readmission status.

Variable	Intervention *N* = 32 *N* (%)	Control *N* = 132 *N* (%)
**Readmission Status**		
Readmitted	3 (9)	17 (13)
Not readmitted	29 (91)	115 (87)
**Readmission Diagnosis**		
Angina	0	2 (12)
Atrial fibrillation	0	1 (6)
Congestive heart failure	1 (33)	1 (6)
Coronary angioplasty	0	1 (6)
Inflammatory disease	0	1 (6)
Medication-related adverse drug event (e.g., bleeding, hypotension, hypoglycemia)	0	3 (17)
Pneumothorax	0	1 (6)
Post-surgery-related infection	1 (33)	0
Renal failure/injury	0	3 (17)
Respiratory	1 (33)	2 (12)

Note: A Fisher’s exact test was used to compare the difference in readmissions. An a priori alpha level of 0.05 was used.

**Table 5:** Differences in MRS post-pharmacist intervention and post-provider review.

Variable	Total *N* = 32	Readmitted *N* = 3	Not Readmitted *N* = 29	*p*-Value
**MRS median (IQR)**				
Pre-pharmacist intervention	14.5 (7.5, 18.5)	19 (8, 21)	13 (7, 18)	0.399
Post-pharmacist intervention	12.5 (7.5, 16)	15 (8, 16)	12 (7, 16.5)	0.897
Post-provider review	12.5 (7.5, 17)	16 (8, 19)	12 (7, 17)	0.559
**Median difference in MRS (IQR)**				
Delta (pre-pharmacist and post-pharmacist intervention)	1 (0, 2)	3 (0, 6)	1 (0, 2)	0.359
Delta (post-pharmacist intervention and post-provider review)	0 (0, 0)	0 (−4, 0)	0 (0, 0)	0.237

Note: A Wilcoxon-rank sum test was used to compare the difference in MRS pre- and post-pharmacist intervention, as well as the difference in MRS post-pharmacist intervention and post-provider review of pharmacist interventions. All tests used an a priori alpha level of 0.05.

## Data Availability

The data presented in this study are available on request from the corresponding author. Primary data are not publicly available as they may contain protected health information and may comply with U.S. Health Insurance Portability and Accountability Act.

## Abbreviations

DDI = drug–drug interaction; MRP = medication-related problem; MRS = medication risk score; MSR = medication safety review; TOC = transitions of care.
